# Pharmacologic intervention for prevention of fractures in osteopenic and osteoporotic postmenopausal women: Systemic review and meta-analysis

**DOI:** 10.1016/j.bonr.2020.100729

**Published:** 2020-10-27

**Authors:** Chih-Hsing Wu, Wei-Chieh Hung, Ing-Lin Chang, Tsung-Ting Tsai, Yin-Fan Chang, Eugene V. McCloskey, Nelson B. Watts, Michael R. McClung, Chun-Feng Huang, Chung-Hwan Chen, Kun-Ling Wu, Keh-Sung Tsai, Ding-Cheng Chan, Jung-Fu Chen, Shih-Te Tu, Jawl-Shan Hwang, Weibo Xia, Toshio Matsumoto, Yoon-Sok Chung, Cyrus Cooper, John A. Kanis, Rong-Sen Yang, Wing P. Chan

**Affiliations:** a Department of Family Medicine, National Cheng Kung University Hospital, College of Medicine, National Cheng Kung University, Tainan, Taiwan; b Institute of Geriatrics, College of Medicine, National Cheng Kung University, Tainan, Taiwan; c Department of Family Medicine, E-Da Hospital/I-Shou University, Kaohsiung, Taiwan; d Institute of Biotechnology and Chemical Engineering, I-Shou University, Kaohsiung, Taiwan; e Medicine for International Student, I-Shou University, Kaohsiung, Taiwan; f Department of Orthopaedics, ChangHua Christian Hospital, ChangHua, Taiwan; g Department of Orthopedics, Chang Gung Memorial Hospital, Chang Gung University, Linkou, Taiwan; h Academic Unit of Bone Metabolism, University of Sheffield, Sheffield, UK; i Mercy Health Osteoporosis and Bone Health Services, Cincinnati, OH, USA; j The Oregon Osteoporosis Center, Portland, OR, USA; k Department of Family Medicine, National Yang Ming University Hospital, I-Lan, Taiwan; l Orthopaedic Research Centre, Kaohsiung Medical University, Kaohsiung, Taiwan; m Department of Orthopaedics, Kaohsiung Medical University Hospital, Kaohsiung Medical University, Kaohsiung, Taiwan; n Department of Orthopaedics, Kaohsiung Municipal Ta-Tung Hospital, Kaohsiung City, Taiwan; o Institute of Medical Science and Technology, National Sun Yat-Sen University, Kaohsiung, Taiwan; p Department of Family Medicine, Tainan Municipal Hospital (Managed by Show Chwan Medical Care Corporation), Tainan City, Taiwan; q Outpatient Clinic Department, Tainan Municipal Hospital (Managed by Show Chwan Medical Care Corporation), Tainan City, Taiwan; r Department of Internal Medicine, National Taiwan University Hospital, Taipei, Taiwan; s Superintendent Office, National Taiwan University Hospital Chu-Tung Branch, Taiwan; t Department of Internal Medicine, National Taiwan University Hospital, Taipei, Taiwan; u Department of Geriatrics and Gerontology, National Taiwan University Hospital, Taipei, Taiwan; v Division of Endocrinology and Metabolism, Department of Internal Medicine, Chang Gung Memorial Hospital, Kaohsiung, Taiwan; w Division of Endocrinology, Department of Internal Medicine, Changhua Christian Hospital, Changhua, Taiwan; x Division of Endocrinology and Metabolism, Department of Internal Medicine, Chang Gung Memorial Hospital, Chang Gung University, Linkou, Taiwan; y Department of Endocrinology, Peking Union Medical College Hospital, Chinese Academy of Medical Sciences, Peking Union Medical College, Beijing, China; z Fujii Memorial Institute of Medical Sciences, University of Tokushima, Japan; aa Department of Endocrinology and Metabolism, Ajou University School of Medicine, Suwon, South Korea; ab MRC Lifecourse Epidemiology Unit, University of Southampton, Southampton, UK; ac Oxford National Institute for Health Research Biomedical Research Centre, University of Oxford, Oxford, UK; ad Mary McKillop Institute for Health Research, Australian Catholic University, Melbourne, Australia; ae Centre for Metabolic Bone Diseases, University of Sheffield Medical School, UK; af Department of Orthopaedics, College of Medicine, National Taiwan University & Hospital, Taipei, Taiwan; ag Department of Radiology, Wan Fang Hospital, Taipei Medical University, Taipei, Taiwan; ah Department of Radiology, School of Medicine, College of Medicine, Taipei Medical University, Taipei, Taiwan

**Keywords:** Fracture, Low bone mass, Osteopenia, Osteoporosis, Primary prevention

## Abstract

**Objectives:**

Emerging evidence has indicated a role for pharmacologic agents in the primary prevention of osteoporotic fracture, but have not yet been systematically reviewed for meta-analysis. We conducted a meta-analysis to evaluate the efficacy of pharmacologic interventions in reducing fracture risk and increasing bone mineral density (BMD) in postmenopausal women with osteopenia or osteoporosis but without prevalent fragility fracture.

**Method:**

The Medline, EMBASE, and CENTRAL databases were searched from inception to September 30, 2019. Only randomized placebo-controlled trials evaluating postmenopausal women with −1.0 > bone mineral density (BMD) T-score > −2.5 (low bone mass) and those with BMD T-score ≤ −2.5 (osteoporosis) but without baseline fractures, who were receiving anti-osteoporotic agents, providing quantitative outcomes data and evaluating risk of vertebral and/or non-vertebral fragility fracture at follow-up. The PRISMA guidelines were followed, applying a random-effects model. The primary endpoint was the effect of anti-osteoporotic regimens in reducing the incidence of vertebral fractures. Secondary endpoints were percentage changes in baseline BMD at the lumbar spine and total hip at 1 and 2 years follow up.

**Results:**

Full-text review of 144 articles yielded, 20 for meta-analysis. Bisphosphonates reduced the risk of vertebral fracture (pooled OR = 0.50, 95%CIs = 0.36–0.71) and significantly increased lumbar spine BMD after 1 year, by 4.42% vs placebo (95%CIs = 3.70%–5.14%). At the hip, this value was 2.94% (95%CIs = 2.13%–3.75%). Overall results of limited studies for non-bisphosphonate drugs showed increased BMD and raloxifene significantly decreases the risk of subsequent clinical vertebral fractures.

**Conclusion:**

The bisphosphonates are efficacious and most evident for the primary prevention of osteoporotic vertebral fractures, reducing their incidence and improving BMD in postmenopausal women with osteopenia or osteoporosis.

## Highlights

• Bisphosphonates reduced the risk of vertebral fracture in postmenopausal women with osteopenia or osteoporosis but without fracture.
• Bisphosphonates increased BMD in postmenopausal women with osteopenia or osteoporosis but without fracture.
• Limited studies for non-bisphosphonate drugs showed increased BMD in postmenopausal women with osteopenia or osteoporosis but without fracture.
• Raloxifene decreased the risk of clinical vertebral fractures in postmenopausal women with osteopenia or osteoporosis but without fracture.

## 1 Introduction

Fragility fractures commonly cause disabilities and mortality in older adults and are major contributors to medical care costs worldwide (Sanchez-Riera and Wilson, 2017). A majority of such fractures occur in postmenopausal women without osteoporosis diagnoses (Lindsay et al., 2001), yet low bone mineral density (BMD) is recognized as an important preventable risk factor for osteoporotic fractures (Sanchez-Riera and Wilson, 2017). Women who develop osteoporotic vertebral and hip fractures are at substantial risk for additional fractures within 1 year (Johansson et al., 2017). Consequently, preventing fragility fractures is of both clinical and public health importance.

Annually, about three million individuals are diagnosed with osteoporosis in the United States (Eriksen, 2012). The U.S. Preventive Services Task Force advises that all women aged more than 65 years should be screened for osteoporosis using dual-energy X-ray absorptiometry (DXA) (USPSTF et al., 2018), which reports results as T-scores based on the standard deviation of the mean BMD in the general population. The World Health Organization defines osteopenia (low bone mass) as a BMD T-score between −1 and − 2.5 at the hip or spine and osteoporosis as a T-score of −2.5 and below (World Health Organization, 2007). The majority of patients presenting with fractures have BMDs in the osteopenic range primarily because the osteopenic population is larger than the osteoporotic population (Eriksen, 2012; Kanis, 1994; Cranney et al., 2007). Although an osteopenic T-score may not indicate a need for treatment, a high risk for future fractures as determined by risk calculators (e.g., FRAX (Siris et al., 2010)) can suggest therapy for osteoporosis (Eriksen, 2012). Theoretically, the intervention for subjects have experienced fracture to prevent a recurrent fracture is considered as “secondary” prevention of osteoporotic fracture. On the contrary, the intervention for subjects with low bone mass or osteoporosis but without clinical fracture is usually called as “primary” prevention of fracture.

The efficacies of various pharmacologic interventions for treating postmenopausal osteoporosis have been compared in many studies, including several noteworthy network meta-analyses (Barrionuevo et al., 2019; Liu et al., 2018; Wang et al., 2017; Yang et al., 2016a; Zhou et al., 2016). Results of such studies have led to a universal acceptance of pharmacologic treatment for osteoporosis with fragility fractures and recommendations by current guidelines (Qaseem et al., 2017). However, fewer studies have addressed the potential efficacy of pharmacologic intervention for the primary prevention of fragility fractures or bone loss in patients with increased risks of fracture due to low bone mass.

Emerging evidence has indicated that pharmacologic agents for osteoporosis treatment, bisphosphonates most abundantly (Iqbal et al., 2019; Grey et al., 2014; Quandt et al., 2005), can also effectively lower fracture risk and prevent bone loss in osteopenic women, as well as patients with T-scores in the osteoporotic range, even those without previous fractures. To date, however, the findings of these and other studies have not been systematically reviewed. Therefore, this review was performed to evaluate the efficacies of pharmacologic interventions on reducing fracture risk and improving BMD in postmenopausal women with osteopenia or osteoporosis but without prior fragility fractures.

## 2 Materials and methods

### 2.1 Search strategy

This study was performed in accordance with PRISMA (Preferred Reporting Items for Systematic Reviews and Meta-Analyses) guidelines. The Medline, EMBASE, and CENTRAL databases were searched from inception to September 30, 2019. The availability of the abstract and the publication language (English) were used as filters in the searches of Medline and EMBASE. Reference lists of relevant studies were manually searched. The following keywords and word combinations were used:

**Table:** 

(osteoporosis OR osteopenia OR low bone mass) AND (alendronate OR alendronic acid OR risedronate OR risedronic acid OR ibandronate OR ibandronic acid OR pamidronate OR pamidronic acid OR zoledronate OR zoledronic acid OR bisphosphonate)
(osteoporosis OR osteopenia OR low bone mass) AND (raloxifene OR bazedoxifene OR SERM OR selective estrogen receptor modulator)
(osteoporosis OR osteopenia OR low bone mass) AND (tibolone OR STEAR OR selective tissue estrogenic activity regulator)
(osteoporosis OR osteopenia OR low bone mass) AND calcitonin
(osteoporosis OR osteopenia OR low bone mass) AND (teriparatide OR parathyroid hormone analog)
(osteoporosis OR osteopenia OR low bone mass) AND (hormone replacement therapy OR estrogen)
(osteoporosis OR osteopenia OR low bone mass) AND strontium ranelate
(osteoporosis OR osteopenia OR low bone mass) AND denosumab OR RANKL inhibitor
(osteoporosis OR osteopenia OR low bone mass) AND romosozumab

### 2.2 Selection criteria

Inclusion criteria were: randomized placebo-controlled trial (RPCT); evaluation of postmenopausal women with low bone mass (or osteopenia or low bone density, defined as −1.0 > BMD T-score > −2.5, measured by DXA) and without baseline vertebral fractures and/or other fragility fractures or of those with osteoporosis (BMD T-score ≤ −2.5) and without baseline vertebral fractures and/or other fragility fractures; pharmacologic intervention for at least 1 year with 1) the nitrogen-containing bisphosphonates alendronate, zoledronate, ibandronate, or risedronate, 2) a selective estrogen receptor modulator (SERM), 3) calcitonin, 4) teriparatide or a parathyroid hormone analog, 5) strontium ranelate, 6) denosumab, 7) estrogen, 8) tibolone, or 9) romosozumab. Studies were also required to have quantitative outcome data evaluating the risk of vertebral and/or non-vertebral fragility fracture at follow-up and/or BMD measures for the lumbar spine or total hip. Cohort studies, letters, comments, reviews, editorials, case reports, proceedings, personal communications, and retrospective studies were excluded. Patients with vertebral fractures at baseline; histories of lumbar spine surgeries; cancer, renal insufficiency, or severe cardiovascular conditions; bone metabolic disease other than osteoporosis, or any illness known to affect bone metabolism (e.g., Paget's disease or hyperparathyroidism); chronic treatment with drugs affecting bone metabolism (eg, glucocorticoids); combination regimens (more than 1 drug) of these drugs; and studies not designed for first-time pharmacologic intervention (ie, inclusion of patients with prior drug therapies) were also excluded.

Studies were identified by 2 independent reviewers, experienced in osteoporosis management, using the above search strategy. When eligibility was uncertain, a third reviewer was consulted.

### 2.3 Data extraction

Data extracted from those studies meeting selection criteria were: name of first author; year of publication; study design; participant group sizes; DXA sites and BMD T-score ranges; treatment protocol (drug, dose, duration, frequency, and route of administration); mean values for participant age, time since menopause, and baseline BMD at the lumbar spine, total hip, and femoral neck; levels of calcium and/or vitamin D supplementation; and outcomes of interest (Table 1 details the independent variables). For trials using various dosages of a single drug, the dosages most similar to other trials were selected to minimize confounding. Various pharmacologic regimens were used, depending on drug: alendronate (70 mg once weekly or 10 mg once daily), zoledronate (5 mg IV infusion), ibandronate (5 mg once daily or 20 mg once weekly or 150 mg once monthly), risedronate (5 mg once daily), teriparatide (20 μg once daily), raloxifene (60 mg daily), denosumab (60 mg every 6 months), or romosozumab (210 mg monthly).

**Table 1:** Characteristics of studies included in the meta-analysis.

First author (year)	Osteopenia/osteoporosis	Therapeutic drug	Time since menopause, y	n. of pts	Treatment protocol			Age, y	Baseline BMD						Combined treatment	
									Lumbar spine		Total hip		Femoral neck			
					Dose	Duration, frequency	Route of administration		g/cm^2^	T-score	g/cm^2^	T-score	g/cm^2^	T-score	Calcium, mg/d	Vitamin D, IU/d
Zoledronate vs placebo																
Grey (2014)	Osteopenia	Zoledronate	n/a	43	5 mg	Single administration	i.v.	66	1.05	−1.1	0.84	−1.3	n/a	n/a	n/a	n/a
		placebo		43				65	1.03	−1.3	0.87	−1.1	n/a	n/a		
Grey (2009)	Osteopenia	Zoledronate	n/a	25	5 mg	Single administration	i.v.	62	1.06	−1.0	0.85	−1.3	n/a	n/a	n/a	n/a
		placebo		25				65	1.03	−1.3	0.86	−1.2	n/a	n/a		
McClung (2009)	Osteopenia	Zoledronate	11.5	198	5 mg	Single administration	i.v.	59.6	0.86	−1.69	0.82	n/a	0.69	−1.40	500–1200	400–800
		placebo	11.4	202				60.5	0.86	−1.71	0.82	n/a	0.69	−1.47		
Reid (2002)	Mixed	Zoledronate	n/a	53	4 mg	Single administration	i.v.	65	0.73	n/a	n/a	n/a	0.74	n/a	1000	n/a
		placebo		57				64	0.74	n/a	n/a	n/a	0.71	n/a		

Alendronate vs placebo																
McClung (2014)	Mixed	Alendronate	n/a	47	70 mg	Once weekly for 1 year either monthly or every 3 months for 1 year	Oral	67.1	n/a	−2.08	n/a	−1.55	n/a	−1.91	1000	800
		Placebo		47			s.c.	67	n/a	−2.29	n/a	−1.35	n/a	−1.76		
McClung (2006)	Mixed	Alendronate	13.7	46	70 mg	Once weekly for 1 year	Oral	62.8	n/a	−2.0	n/a	−1.6	n/a	−1.9	n/a	n/a
		placebo	13.8	46			Oral	63.7	n/a	−2.2	n/a	−1.4	n/a	−1.9	n/a	n/a
Quandt (2005)	Osteopenia	Alendronate	20.2	1394	5 mg daily for the first 2 years then 10 mg daily		Oral	66.9	0.87	n/a	0.75	n/a	n/a	n/a	500	250
		Placebo	20.4	1403				67	0.88	n/a	0.74	n/a	n/a	n/a		
Ascott-Evans (2003)	Mixed	Alendronate	11.4	95	10 mg	Once daily for 1 year	Oral	57.3	−2.30	n/a	n/a	n/a	n/a	n/a	500	n/a
		Placebo	11.6	49				57.3	−2.22	n/a	n/a	n/a	n/a	n/a		
Downs (2000)	Mixed	Alendronate	16.5	118	10 mg	Once daily for 1 year	Oral	64.6	n/a	−2.54	n/a	n/a	n/a	−2.63	500	400
		Placebo	16.5	58			Oral	64.6	n/a	−2.36	n/a	n/a	n/a	−2.59		
Yen (2000)	Mixed	Alendronate	11.7	24	10 mg	Once daily for 1 year	Oral	59	0.72	−1.9				−1.9	500	n/a
		Placebo	11.8	22				60.3	0.72	−1.9				−1.6		
Cummings (1998)	Mixed	Alendronate	n/a	2214	5 mg daily for the first 2 years then 10 mg daily		Oral	67.6	0.841	n/a	n/a	n/a	0.592	n/a	500	250
		Placebo		2218				67.7	0.842	n/a	n/a	n/a	0.593	n/a		

Ibandronate vs placebo																
Bock (2011)	Mixed	Ibandronate	19.3	35	150 mg	Once monthly for 1 year	Oral	69.3	0.76	−2.6	0.82	−1.0	n/a	n/a	500	400
		Placebo	19.1	33				68.6	0.77	−2.5	0.84	−0.9	n/a	n/a		
Ravn (1996)	Mixed	Ibandronate	n/a	18	5.0 mg	Once daily for 1 year	Oral	64.4	0.861	n/a	n/a	n/a	0.665	n/a	1000	n/a
		Placebo		25				64.4	0.826	n/a	n/a	n/a	0.653	n/a		

Risedronate vs placebo																
Siris (2008)	Osteopenia	Risedronate	n/a	311	5 mg	Once daily for 1.5 to 3 years	Oral	64	n/a	n/a	n/a	n/a	n/a	−1.85	1000	500
		Placebo		309				64	n/a	n/a	n/a	n/a	n/a	−1.84		

Abbreviations: i.v., intravenous infusion; s.c., subcutaneous injection; n/a, not available.

### 2.4 Ethics statement

The protocol for this review and meta-analysis was reviewed and approved by the Internal Review Board of National Cheng Kung University Hospital (A-EX-108-051). Raw patient data and private information were neither required nor used; therefore, informed consent was unnecessary.

### 2.5 Quality assessment

The Cochrane Collaboration tool (Higgins et al., 2011) was used to assess included studies for risk of bias via seven criteria: selection bias (random sequence generation; allocation concealment), performance bias (blinding of participants and personnel), detection bias (blinding of outcome assessment), attrition bias (incomplete outcome), reporting bias (selective outcome reporting), and inclusion of intention-to-treat analysis. Quality assessment was performed by 2 independent reviewers, consulting a third reviewer in the case of uncertainty.

### 2.6 Outcome measures

The primary endpoint for this meta-analysis was the effect of bisphosphonates in reducing the incidence of vertebral fractures. Secondary endpoints were percentage changes between the baseline BMD at the lumbar spine and/or total hip and after 1 and 2 years of follow-up. Fracture risk reduction was measured as the difference between the treated and placebo groups in the incidence of vertebral fractures during follow-up and improvement in BMD at the lumbar spine and total hip as measured by the mean percentage change between baseline values and those at 1 and/or 2 years of follow-up.

### 2.7 Statistical analysis

A χ^2^-based test of homogeneity was performed using Cochrane's Q statistic and *I*^2^, the percentage of total variability in effect estimates among included trials that results from heterogeneity rather than chance. A random-effects model was also applied. Odds ratios (ORs) were calculated for the primary endpoint using 2 × 2 tables, and additional ORs (with corresponding 95% confidence intervals [CIs]) were calculated for combined studies. Differences in means (with corresponding 95% CIs) were calculated for the secondary endpoints for each individual study and for combined studies. Pooled effects were calculated, and a 2-tailed *P* value < 0.05 was established for statistical significance. Subgroup analyses of treatment effects were performed for each specific bisphosphonate (zoledronate, alendronate, ibandronate, and risedronate) when the BMD T-score indicated osteopenia.

A sensitivity analysis was carried out using the leave-one-out approach. Publication bias was evaluated using funnel plots (Sterne et al., 2011) assessed by applying the Egger's test for asymmetry. The absence of publication bias was found when the data points formed a symmetric funnel-shaped distribution, and the one-tailed significance level was *P* > 0.05. All analyses were performed using Comprehensive Meta-Analysis statistical software, version 2.0 (Biostat, Englewood, NJ, USA).

## 3 Results

### 3.1 Search results

The PRISMA flow diagram for study selection is shown in Supplemental Fig. 1. Of 144 articles identified for full-text review, 124 were excluded, and the 20 meeting inclusion criteria were included this study (Grey et al., 2014; Quandt et al., 2005; Ascott-Evans et al., 2003; Binkley et al., 2014; Bock et al., 2012; Bone et al., 2008; Cummings et al., 1998; Downs Jr. et al., 2000; Grey et al., 2009; Ishibashi et al., 2017; Kanis et al., 2003; McClung et al., 2006; McClung et al., 2009; McClung et al., 2014; Naylor et al., 2016; Ravn et al., 1996; Reid et al., 2002; Siris et al., 2008; Yang et al., 2016b; Yen et al., 2000). Of those, 14 focused on the effects of bisphosphonates and were included in the meta-analysis (quantitative analysis), and 9 focused on non-bisphosphonate drugs (Binkley et al., 2014; Bone et al., 2008; Downs Jr. et al., 2000; Ishibashi et al., 2017; Kanis et al., 2003; McClung et al., 2006; Naylor et al., 2016; Yang et al., 2016b) and were evaluated qualitatively only (3 RPCTs (Downs Jr. et al., 2000; McClung et al., 2006; McClung et al., 2014) evaluated bisphosphonates and non-bisphosphonate drugs; results were included in both the meta-analysis and the qualitative evaluation).

### 3.2 Study characteristics

The primary characteristics of those studies included in the meta-analysis are summarized in Table 1. In total, the 14 studies reported results for 8813 postmenopausal women, 4500 receiving bisphosphonates and 4313 receiving placebo, who met the criteria for low bone mass based on BMD T-scores and lacked baseline vertebral fractures. In total, 319 women were treated with zoledronate, 3938 with alendronate, 153 with ibandronate, and 311 with risedronate. Mean participant age ranged from 57.3 to 69.3 years. Study characteristics for those focusing on non-bisphosphonate pharmacologic agents are shown in Table 2.

**Table 2:** Characteristics of the studies on non-bisphosphonate drugs.

First author (year)	Osteopenia/osteoporosis	Therapeutic drug	Time since menopause, y	No. of pts	Treatment protocol			Age, y	Baseline BMD						Combined treatment	
									Lumbar spine		Total hip		Femoral neck			
					Dose	Duration, frequency	Route of administration		g/cm^2^	T-score	g/cm^2^	T-score	g/cm^2^	T-score	Calcium, mg/d	Vitamin D, IU/d
Ishibashi (2017)	Osteoporosis	Romosozumab	n/a	59	210 mg	Once monthly for 1 year	s.c.	68.3	n/a	−2.72	n/a	−1.95	n/a	−2.32	≥500	≥600
		Placebo		59				67.8	n/a	−2.69	n/a	−2.00	n/a	−2.31		
Naylor (2016)	Osteopenia	Raloxifene	n/a	21	60 mg	Once daily for 1 year	Oral	63	0.857	n/a	n/a	n/a	n/a	n/a	500	n/a
		control (no treatment)		23				61	0.903	n/a		n/a	n/a			
Yang (2016)	Osteopenia	Teriparatide	14.5		20 μg	Once daily for 1 year	s.c.	64.3	1.003	n/a	0.902	n/a	0.805		1000	400
		Placebo	13.6				Oral	63.9	1.01	n/a	0.906	n/a	0.801			
Binkley (2014)	Osteopenia	Calcitonin	≥5	78	0.2 mg	Once daily for 1 year	Oral	67.5	n/a	−1.15	n/a	−1.23	n/a	−1.69	600	1000
		Placebo		36				66.6	n/a	−1.12		−1.20	n/a	−1.73		
McClung (2014)	Mixed	Teriparatide	n/a	46	20 μg	Once daily for 1 year	s.c.	66.8	n/a	−2.29	n/a	−1.32	n/a	−1.79	1000	800
		Romosozumab		49	210 mg	Once monthly for 1 year		66.3	n/a	−2.33	n/a	−1.45	n/a	−1.87		
		Placebo		47		Either monthly or every 3 months for 1 year		67	n/a	−2.29	n/a	−1.35	n/a	−1.76		
Bone (2008)	Osteopenia	Denosumab	10.5	166	60 mg	Every 6 months for 2 years (at months 6, 12, and 18)	s.c.	59.8	n/a	−1.55	n/a	n/a	n/a	n/a	1000	400–800
		Placebo	9.4	166			oral	58.9	n/a	−1.66	n/a	n/a	n/a	n/a		
McClung (2006)	Mixed	Denosumab	15.7	46	60 mg		Oral		n/a	−2.2	n/a	−1.4	n/a	−1.9	1000	400
		Placebo	13.8	46				63.7	n/a	−2.2	n/a	−1.4	n/a	−1.9		
Kanis (2003)	Osteopenia	Raloxifene	n/a	1287	60 mg	Once daily for 3 years	Oral	65.2	0.85	−2.28	0.75	−1.71	0.63	−2.12	500	400–600
		Placebo		1270												
	Osteoporosis	Raloxifene		285	60 mg	Once daily for 3 years	Oral	66.2	0.76	−3.07	0.61	−2.86	0.63	−2.85		
		placebo		350												
Downs (2000)	Mixed	Calcitonin	16.1	123	200 IU	Once daily for 1 year	Intra-nasal	64.1	n/a	−2.54	n/a	n/a	n/a	−2.71	500	400
		Placebo	16.5	58			Oral	64.6	n/a	−2.36	n/a	n/a	n/a	−2.59		

Abbreviations: s.c., subcutaneous injection; n/a, not applicable.

### 3.3 Meta-analyses

#### 3.3.1 Vertebral fractures

**Fig. 1:**
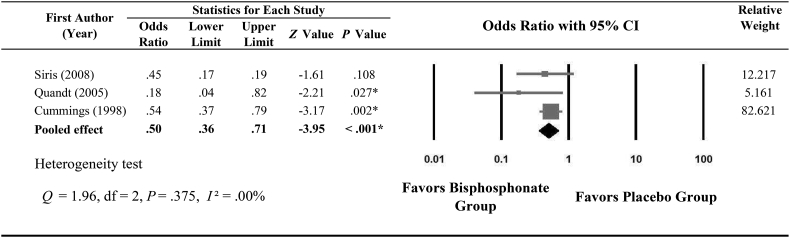
Meta-analysis of the effect of bisphosphonate on reducing vertebral fractures in postmenopausal women with osteopenia/osteoporosis.

Only 3 studies (Quandt et al., 2005; Cummings et al., 1998; Siris et al., 2008) providing data about vertebral fractures were included in the meta-analysis. Fig. 1 shows the forest plot indicating the effect of bisphosphonates on reducing vertebral fractures in postmenopausal women. Heterogeneity was not found between these 3 studies (*Q* = 1.96; *I*^2^ = 0.00%, *P* = 0.375). Results of the meta-analysis showed that bisphosphonates reduce the risk of subsequent vertebral fracture by 50% (pooled OR, 0.50; 95% CI, 0.36–0.71; *P* < 0.001). From the Fig. 1, the experimental event rate (EER) is 0.014, the control event rate (CER) is 0.028, the absolute risk reduction (ARR) is 0.014 and NNT (number-needed to treat) is 71. That is, 71 patients would have to receive bisphosphonate treatment (instead of placebo) for one additional patient to not have vertebral fracture.

#### 3.3.2 Bone mineral density at the lumbar spine and total hip after 1 year of follow-up

**Fig. 2:**
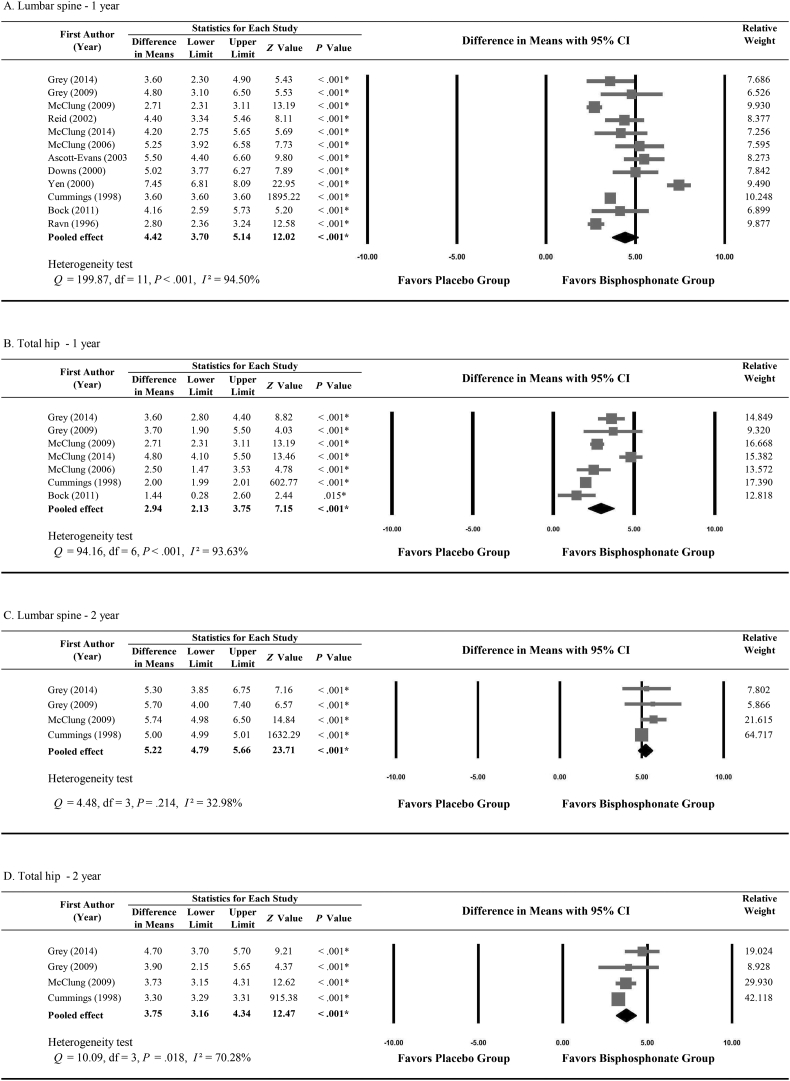
Bone mineral density differences between bisphosphonate and placebo groups. Differences after 1 year of follow up at A, the lumbar spine and B, the total hip. Differences after 2 years of follow up at C, the lumbar spine and D, the total hip.

Twelve studies provided adequate data about the percentage change in BMD at the lumbar spine between baseline and 12 months of follow-up. Fig. 2A shows how the change in BMD at the lumbar spine differed between the bisphosphonate and placebo groups. Significant heterogeneity was found among the 12 studies (*Q* = 199.87; *I*^2^ = 94.50%, *P* < 0.001). Results show that bisphosphonates significantly increased lumbar spine BMD after 1 year, and the difference in mean BMD change versus placebo was 4.42% (95% CI, 3.70%–5.14%).

Seven studies (Grey et al., 2014; Bock et al., 2012; Cummings et al., 1998; Grey et al., 2009; McClung et al., 2006; McClung et al., 2009; McClung et al., 2014) provided adequate data about the percentage change in BMD in the total hip between baseline and 1 year of follow-up. Fig. 2B shows how the change in BMD in the total hip differed between the bisphosphonate and placebo groups. Significant heterogeneity was found among the 7 studies (*Q* = 94.16; *I*^2^ = 93.63%, *P* < 0.001). Results show a 2.94% difference in BMD changes between the bisphosphonate and placebo groups (95% CI, 2.13%–3.75%; *P* < 0.001).

#### 3.3.3 Bone mineral density at the lumbar spine and total hip after 2 years of follow-up

Only 4 studies (Grey et al., 2014; Cummings et al., 1998; Grey et al., 2009; McClung et al., 2009) provided adequate data about the percentage change in BMD at the lumbar spine or total hip after 2 years of follow-up. Results show that after 2 years, the bisphosphonate groups had greater increases in BMD at the lumbar spine and total hip, besting the placebo groups by 5.22% (95% CI, 4.79%–5.66%; *P* < 0.001; Fig. 2C) and 3.75% (95% CI, 3.16%–4.34%; *P* < 0.001; Fig. 2D), respectively.

### 3.4 Sensitivity analysis and publication bias

A sensitivity analysis was performed using the leave-one-out approach (ie, meta-analysis was repeated, removing each study in turn). Results are shown in Supplemental Table 1. The direction and magnitude of the combined estimates did not vary markedly with the removal of any given study, indicating good reliability in the meta-analysis and that the result was not overly influenced by any individual study.

The Egger's test showed that publication bias was not present in the BMD results at the lumbar spine after 1 year of follow-up (*t* = 1.179, *P* = 0.133; Supplemental Fig. 1).

### 3.5 Subgroup analysis

Table 3 shows the results of subgroup analyses exploring the treatment effectiveness of each individual bisphosphonate drug. Zoledronate, after 1 year, increased BMD more than placebo by 3.74% at the lumbar spine and 3.15% in the total hip. Results were similar after 1 year of treatment with alendronate (5.18% and 3.09%, respectively) and after treatment with ibandronate (3.26% and 1.44%, respectively).

**Table 3:** Summary effects of bisphosphonates versus placebo on BMD by subgroup.

	Number of studies	*Q* statistic	*I*^2^	Pooled effect size with 95% CI	*P* value
Lumbar spine — 1 year					
All women					
Zoledronate vs placebo	4	13.68	78%	3.74	<0.001
Alendronate vs placebo	6	163.61	97%	5.18	<0.001
Ibandronate vs placebo	2	2.68	63%	3.26	<0.001
Osteopenia subgroup					
Bisphosphonate vs placebo	3	2.76	70%	3.48	<0.001

Total hip — 1 year					
All women					
Zoledronate vs placebo	3	4.54	56%	3.15	<0.001
Alendronate vs placebo	3	62.55	97%	3.09	0.001
Ibandronate vs placebo	1	NA	n/a	1.44	0.015
Osteopenia subgroup					
Bisphosphonate vs placebo	3	4.54	56%	3.15	<0.001

Lumbar spine — 2 year					
All women					
Zoledronate vs placebo	3	0.28	0%	5.65	<0.001
Alendronate vs placebo	1	NA	n/a	5	<0.001
Osteopenia subgroup					
Bisphosphonate vs placebo	3	0.28	0%	5.65	<0.001

Total hip — 2 year					
All women					
Zoledronate vs placebo	3	2.71	26%	4.04	<0.001
Alendronate vs placebo	1	NA	n/a	3.3	0.001
Osteopenia subgroup					
Bisphosphonate vs placebo	3	2.71	26%	4.04	<0.001

Abbreviations: BMD, bone mineral density; CI, confidence interval; n/a, not applicable.

After 2 years of treatment, zoledronate increased BMD more than placebo by 5.65% and 4.04% at the lumbar spine and in the total hip, respectively.

When baseline BMD T-scores indicated osteopenia (−1 > T-score > −2.5), bisphosphonates led to significantly greater increases in BMD after 1 and 2 years compared to placebo at both locations.

### 3.6 Non-bisphosphonate drugs

A qualitative review of non-bisphosphonate drugs was performed using the 9 studies of post-menopausal women (mean age, 58.9 to 68.3 years) that met inclusion criteria (see Table 2). Three studies (Downs Jr. et al., 2000; McClung et al., 2006; McClung et al., 2014) included both bisphosphonate and non-bisphosphonate drugs and subsequently appear in both Table 1, Table 2. Overall, the non-bisphosphonate drugs were promising for increasing BMD in postmenopausal women with low bone mass at baseline.

#### 3.6.1 Denosumab

After 12 months' intervention, BMD significantly increased at the lumbar spine (3.0%–6.7%) and the total hip (1.9%–3.6%) compared to placebo (−0.8% and −0.6%, respectively) in postmenopausal women with low bone densities (McClung et al., 2006). Given biannually, denosumab significantly increased BMD at the lumbar spine compared to placebo (6.5% vs −0.6%) in women with lumbar spine BMD T-scores between −1.0 and −2.5 (Bone et al., 2008).

#### 3.6.2 Romosozumab

After 12 months' treatment, BMD significant increased in Japanese postmenopausal women compared to placebo (Ishibashi et al., 2017). At all dosages, romosozumab significantly increased BMD compared to placebo. At the lumbar spine and after 12 months of treatment, the 210-mg monthly dose increased BMD 11.3% compared to −0.1% with placebo in postmenopausal women with low bone density (McClung et al., 2014).

#### 3.6.3 Raloxifene

At week 48, the change in spine BMD was significantly different in the treatment group (0.031 g/cm^2^) compared to the no-treatment group (2.0% vs −1.1%) in postmenopausal osteopenic women (Naylor et al., 2016). In 3204 osteopenic and osteoporotic women without vertebral fractures at baseline, the 60-mg daily dose significantly decreased the risk of subsequent clinical vertebral fractures compared to the placebo group (relative fracture risk, 0.25) (Kanis et al., 2003).

#### 3.6.4 Teriparatide

A 20-μg daily subcutaneous dose resulted in increased BMD at the lumbar spine compared to placebo (7.1% vs −0.1%) after 12 months of treatment (McClung et al., 2014). This regimen also resulted in mean changes in BMD of 3.51%, 2.10%, and 1.80% at the lumbar spine, total hip, and femoral neck, respectively, in postmenopausal osteopenic women (Yang et al., 2016b).

#### 3.6.5 Calcitonin

Treatment-naïve postmenopausal women with low bone densities and who received oral salmon calcitonin once daily for 1 year showed significant increases between baseline BMD and 24- and 58-week follow-ups (0.96% and 1.03%, respectively). Compared to placebo, these differences are significant (Binkley et al., 2014). Intranasal calcitonin therapy in osteoporotic postmenopausal women without prevalent vertebral fractures, changes in BMD at the femoral neck were greater than those with placebo, but this was not true for the lumbar spine BMD at 12 months (Downs Jr. et al., 2000).

### 3.7 Quality assessment

Results of the quality assessment for the included studies are shown in Supplemental Fig. 2. The summary of assessment indicated that the included studies could have a slight risk for bias and fair application concerns (Supplemental Fig. 2A) but all demonstrated adequate quality (Supplemental Fig. 2B).

## 4 Discussion

Results of this meta-analysis show that nitrogen-containing bisphosphonates are efficacious in the primary prevention of vertebral fracture in postmenopausal women with osteopenia or osteoporosis, reducing the risk of fracture by approximately 50%. Nitrogen-containing bisphosphonates are also efficacious in improving BMD at the lumbar spine or total hip after 1 to 2 years of follow-up, even in osteopenic patients. The specific bisphosphonate drugs, zoledronate, alendronate, and ibandronate, each showed significant benefits in improving BMD. The evidence gathered during this qualitative review indicate that the non-bisphosphonate agents denosumab, romosozumab, raloxifene, teriparatide, and calcitonin are also efficacious in primary prevention, but meta-analysis was not possible with the small number of studies.

Our results are comparable to those of a number of RPCTs, yet this work was more comprehensive and clearly defined than some other studies. To date, only 1 other systematic review shared our goal and focused on the use of bisphosphonates in osteopenia rather than exploring osteoporosis (Iqbal et al., 2019). However, meta-analyses were not performed in that study, and baseline fragility fractures were not ruled out, as in this study. A recent double-blind RPCT that investigated 2000 osteopenic women who received various doses of infusions of the most potent bisphosphonate, zoledronate (zoledronic acid), showed that vertebral and nonvertebral fragility fractures were significantly lower at 6-year follow-up (Reid et al., 2018). Those authors found, as we did, that bisphosphonates clearly reduce fracture risk and result in an incremental improvement in BMD in those with osteopenia. However, in that trial, women with pre-existing fractures were not excluded (nonvertebral fractures were seen in 24% of the participants at screening). The authors noted that, on the basis of their sub-analyses, when excluding the women with BMD T-scores less than −2, those with baseline risks of hip fracture exceeding 3%, those with baseline risks of major osteoporotic fracture exceeding 20% according to FRAX, and those with histories of nonvertebral fracture after 45 years old, zoledronate reduced total fragility fractures by 40% and non-vertebral fragility fractures by 43% (Reid et al., 2018).

Unfortunately, because the data were insufficient, the summary effects for the efficacy of bisphosphonates on non-vertebral fractures could not be determined. Nevertheless, some included studies reported the incidence of non-vertebral fractures between treatment and placebo groups and included any decreased risk of non-vertebral fractures (relative hazard, 0.88) after 4 years of alendronate therapy versus placebo (Cummings et al., 1998). Additionally, a significant reduction in the risk for non-vertebral fractures (relative hazard, 0.09) was found when comparing 3 years of risedronate therapy with placebo across over 600 women with osteopenia (Siris et al., 2008).

In most of the included studies, changes in BMD between baseline and follow-up were evaluated at 1 or 2 years. However, extended follow-up results were sometimes reported, offering additional insight. In the Fracture Intervention Trial, Cummings et al. (Cummings et al., 1998) tested the hypothesis that 4 years of alendronate therapy would decrease fracture risks in women with low bone densities but without vertebral fractures; the finding was that BMD was safely increased across 4 consecutive years, and the risk of a first vertebral deformity also was decreased. This study followed another by the same authors which showed that alendronate decreased the risks of vertebral, hip, and wrist fractures by 50% and clinical fractures by 28% in women with prior vertebral fractures. McNabb et al. (McNabb et al., 2013) studied patterns of change in BMD to determine whether rates of BMD loss changed after discontinuing bisphosphonate therapy for 3 to 5 years. Results showed that one-third of women who discontinued bisphosphonate therapy had more than 5% bone loss in the total hip after 5 years; age and body mass index were risk factors for greater bone loss after drug discontinuance. Grey et al. (Grey et al., 2017) extended their 2-year RPCT by an additional 3 years (Grey et al., 2009) to achieve 5 years of total therapy and explore the duration of antiresorptive activity of zoledronate in postmenopausal women with osteopenia. Results showed that a single dose of zoledronate produced a persistent antiresorptive effect (Grey et al., 2017).

Though pooled estimates for non-bisphosphonates was not possible in our work, we qualitatively reviewed the studies and found that non-bisphosphonate agents might also be effective in the primary prevention of fragility fractures. Kanis et al. (Kanis et al., 2003) showed that raloxifene significantly decreased the risk of subsequent clinical vertebral fractures in osteopenic or osteoporotic women without prior vertebral fractures. Compared to the no-treatment group, the treatment group showed a significant change in spine BMD at week 48 in postmenopausal osteopenic women (Naylor et al., 2016). We found that after 12 months of denosumab, BMD increased 3.0% to 6.7% at the lumbar spine and 1.9% to 3.6% in the total hip (McClung et al., 2006). Also, twice-yearly injectable denosumab increased BMD at the lumbar spine (Bone et al., 2008). Recent studies compared bisphosphonates with various agents in osteoporotic women with/without prior fragility fractures (Lyu et al., 2019; Wu et al., 2018). Lyu et al. (Eriksen, 2012) found that denosumab increased BMD more than bisphosphonates at the lumbar spine, total hip, and femoral neck at 12 and 24 months, leading to a lower incidence of osteoporotic fractures at 24 months (overall meta-analysis, not limited to patients with osteopenia). Another meta-analysis evaluating denosumab vs bisphosphonates showed that denosumab, but not bisphosphonates, significantly increased BMD at the lumbar spine, total hip, and femoral neck in postmenopausal osteoporosis patients (Wu et al., 2018). In an indirect comparison of denosumab, teriparatide, and oral bisphosphonates, Zhang et al. (2015) demonstrated that teriparatide and denosumab were more effective than alendronate and risedronate in reducing vertebral fractures in osteoporotic women. Clearly, future studies for evaluating whether these drugs are more efficacious than bisphosphonates for the primary prevention for fractures among women with low bone densities are warranted.

Our review showed that mean differences in BMD change ranged from 5% to 6% between the treatment and placebo groups. In a recent meta-regression of 38 placebo-controlled trials of 19 therapeutic agents, Bouxsein (Bouxsein et al., 2019) concluded that a 2% to 6% improvement in total hip BMD implies a 28% to 66% reduction in risk for vertebral fracture. Changes in BMD noted in individual-level patient data from the FNIH Bone Quality Project supports this ratio between BMD improvement and fracture risk reduction (Black and Eastell, 2018). Post-treatment changes in BMD at 12 or 24 months, especially in the hip, serve as the most important surrogate endpoints for evaluating vertebral and hip fracture risk reduction and therapeutic response (Bouxsein et al., 2019; Black and Eastell, 2018). Even small increases in BMD after treatment can be associated with a considerable reduction in subsequent fracture risk and could translate into clinically important fracture differences when observed in large populations. Accordingly, our post-treatment BMD results are clinically relevant.

### 4.1 Strengths and limitations

This meta-analysis evaluated the efficacy of pharmacological intervention for the primary prevention of fragility fractures in patients with osteopenia. Importantly, the inclusion and exclusion criteria were rigorously established, and the outcomes were carefully defined.

Furthermore, all included studies were RPCTs of high quality. Pooled estimates of ORs were generally without high CIs, suggesting satisfactory accuracy and reliability of the findings. Subgroup analyses were also conducted to identify potential bias, and a sensitivity analysis revealed the robustness of the primary results.

Nevertheless, this review and meta-analysis has several limitations. The summary effects for non-vertebral fractures, total hip BMDs, and femoral neck BMDs could not be obtained due to lack of data. Because the presence of prior fragility fractures in many studies depended on self-reported medical histories, those with subclinical fragility fractures might have been erroneously included. Therefore, only those studies with clear descriptions of prior fragility fractures or spinal radiography were enrolled. The number of studies that analyzed vertebral fracture was also limited, though heterogeneity was low. The analysis of non-bisphosphonate drugs was performed as a qualitative review only. Treatment duration, changes in bone markers, and adverse events from pharmacologic interventions could not be assessed due to insufficient data across studies. Future studies must include comparative efficacy analyses and cost-effectiveness analyses of various pharmacologic interventions for the primary prevention of osteoporotic fracture.

## 5 Conclusions

Of all the anti-osteoporotic regimens, the bisphosphonates are efficacious and more evident for the prevention of osteoporotic fracture, for reducing the incidence of osteoporotic vertebral fractures, and for supporting improvements in BMD values in postmenopausal women with osteopenia or osteoporosis without fracture. Additional evidence is necessary to strengthen the efficacy of non-bisphosphonate regimens in the prevention of osteoporotic fractures in postmenopausal women with osteopenia or osteoporosis without fracture.

The following are the supplementary data related to this article.

Supplemental Fig. 1 PRISMA flow diagram for study selection. Supplemental Fig. 1

Supplementary material 1 Image 1

Supplementary material 2 Image 2

## Transparency document

Transparency document. Image 3

## CRediT authorship contribution statement

**Chih-Hsing Wu:** Methodology, Investigation, Data curation, Writing - original draft, Formal analysis, Validation. **Wei-Chieh Hung:** Methodology, Investigation, Data curation, Writing - original draft, Formal analysis, Validation. **Ing-Lin Chang:** Methodology, Validation. **Tsung-Ting Tsai:** Methodology, Validation. **Yin-Fan Chang:** Writing - review & editing, Validation. **Eugene V. McCloskey:** Investigation, Writing - review & editing, Validation. **Nelson B. Watts:** Investigation, Writing - review & editing, Validation. **Michael R. McClung:** Investigation, Writing - review & editing, Validation. **Chun-Feng Huang:** Writing - review & editing, Validation. **Chung-Hwan Chen:** Writing - review & editing, Validation. **Kun-Ling Wu:** Writing - review & editing, Validation. **Keh-Sung Tsai:** Writing - review & editing, Validation. **Ding-Cheng Chan:** Writing - review & editing, Validation. **Jung-Fu Chen:** Writing - review & editing, Validation. **Shih-Te Tu:** Writing - review & editing, Validation. **Jawl-Shan Hwang:** Writing - review & editing, Validation. **Weibo Xia:** Writing - review & editing, Validation. **Toshio Matsumoto:** Writing - review & editing, Validation. **Yoon-Sok Chung:** Writing - review & editing, Validation. **Cyrus Cooper:** Investigation, Writing - review & editing, Validation. **John A. Kanis:** Investigation, Writing - review & editing, Validation. **Rong-Sen Yang:** Writing - review & editing, Validation. **Wing P. Chan:** Formal analysis, Writing - review & editing, Validation.

## Declaration of competing interest

The authors declare that they have no known competing financial interests or personal relationships that could have appeared to influence the work reported in this paper.

## References

[bb0005] Ascott-Evans B.H., Guanabens N., Kivinen S., Stuckey B.G., Magaril C.H., Vandormael K. (2003). Alendronate prevents loss of bone density associated with discontinuation of hormone replacement therapy: a randomized controlled trial. Arch. Intern. Med..

[bb0010] Barrionuevo P., Kapoor E., Asi N., Alahdab F., Mohammed K., Benkhadra K. (2019). Efficacy of pharmacological therapies for the prevention of fractures in postmenopausal women: a network meta-analysis. J. Clin. Endocrinol. Metab..

[bb0015] Binkley N., Bone H., Gilligan J.P., Krause D.S. (2014). Efficacy and safety of oral recombinant calcitonin tablets in postmenopausal women with low bone mass and increased fracture risk: a randomized, placebo-controlled trial. Osteoporos. Int..

[bb0020] Black D.V.E., Eastell R. (2018). Change in BMD as a surrogate for fracture risk reduction in osteoporosis trials: results from pooled, individual-level patient data from the FNIH Bone Quality Project. Annual Meeting of the American Bone and Mineral Research Society, Montreal, Quebec, Canada.

[bb0025] Bock O., Borst H., Beller G., Armbrecht G., Degner C., Martus P. (2012). Impact of oral ibandronate 150 mg once monthly on bone structure and density in post-menopausal osteoporosis or osteopenia derived from in vivo muCT. Bone..

[bb0030] Bone H.G., Bolognese M.A., Yuen C.K., Kendler D.L., Wang H., Liu Y. (2008). Effects of denosumab on bone mineral density and bone turnover in postmenopausal women. J. Clin. Endocrinol. Metab..

[bb0035] Bouxsein M.L., Eastell R., Lui L.Y., Wu L.A., de Papp A.E., Grauer A. (2019). Change in bone density and reduction in fracture risk: a meta-regression of published trials. J. Bone Miner. Res..

[bb0040] Cranney A., Jamal S.A., Tsang J.F., Josse R.G., Leslie W.D. (2007). Low bone mineral density and fracture burden in postmenopausal women. CMAJ..

[bb0045] Cummings S.R., Black D.M., Thompson D.E., Applegate W.B., Barrett-Connor E., Musliner T.A. (1998). Effect of alendronate on risk of fracture in women with low bone density but without vertebral fractures: results from the Fracture Intervention Trial. JAMA..

[bb0050] Downs R.W., Bell N.H., Ettinger M.P., Walsh B.W., Favus M.J., Mako B. (2000). Comparison of alendronate and intranasal calcitonin for treatment of osteoporosis in postmenopausal women. J. Clin. Endocrinol. Metab..

[bb0055] Eriksen E.F. (2012). Treatment of osteopenia. Rev. Endocr. Metab. Disord..

[bb0060] Grey A., Bolland M.J., Wattie D., Horne A., Gamble G., Reid I.R. (2009). The antiresorptive effects of a single dose of zoledronate persist for two years: a randomized, placebo-controlled trial in osteopenic postmenopausal women. J. Clin. Endocrinol. Metab..

[bb0065] Grey A., Bolland M., Mihov B., Wong S., Horne A., Gamble G. (2014). Duration of antiresorptive effects of low-dose zoledronate in osteopenic postmenopausal women: a randomized, placebo-controlled trial. J. Bone Miner. Res..

[bb0070] Grey A., Bolland M.J., Horne A., Mihov B., Gamble G., Reid I.R. (2017). Duration of antiresorptive activity of zoledronate in postmenopausal women with osteopenia: a randomized, controlled multidose trial. CMAJ.

[bb0075] Higgins J.P., Altman D.G., Gotzsche P.C., Juni P., Moher D., Oxman A.D. (2011). The Cochrane Collaboration's tool for assessing risk of bias in randomised trials. BMJ..

[bb0080] Iqbal S.M., Qamar I., Zhi C., Nida A., Aslam H.M. (2019). Role of bisphosphonate therapy in patients with osteopenia: a systemic review. Cureus..

[bb0085] Ishibashi H., Crittenden D.B., Miyauchi A., Libanati C., Maddox J., Fan M. (2017). Romosozumab increases bone mineral density in postmenopausal Japanese women with osteoporosis: a phase 2 study. Bone..

[bb0090] Johansson H., Siggeirsdottir K., Harvey N.C., Oden A., Gudnason V., McCloskey E. (2017). Imminent risk of fracture after fracture. Osteoporos. Int..

[bb0095] Kanis J.A. (1994). Assessment of fracture risk and its application to screening for postmenopausal osteoporosis. Report of a WHO Study Group. World Health Organ. Tech. Rep. Ser..

[bb0100] Kanis J.A., Johnell O., Black D.M., Downs R.W., Sarkar S., Fuerst T. (2003). Effect of raloxifene on the risk of new vertebral fracture in postmenopausal women with osteopenia or osteoporosis: a reanalysis of the Multiple Outcomes of Raloxifene Evaluation trial. Bone.

[bb0105] Lindsay R., Silverman S.L., Cooper C., Hanley D.A., Barton I., Broy S.B. (2001). Risk of new vertebral fracture in the year following a fracture. JAMA..

[bb0110] Liu G.F., Wang Z.Q., Liu L., Zhang B.T., Miao Y.Y., Yu S.N. (2018). A network meta-analysis on the short-term efficacy and adverse events of different anti-osteoporosis drugs for the treatment of postmenopausal osteoporosis. J. Cell. Biochem..

[bb0115] Lyu H., Jundi B., Xu C., Tedeschi S.K., Yoshida K., Zhao S. (2019). Comparison of denosumab and bisphosphonates in patients with osteoporosis: a meta-analysis of randomized controlled trials. J. Clin. Endocrinol. Metab..

[bb0120] McClung M.R., Lewiecki E.M., Cohen S.B., Bolognese M.A., Woodson G.C., Moffett A.H. (2006). Denosumab in postmenopausal women with low bone mineral density. N. Engl. J. Med..

[bb0125] McClung M.R., Miller P., Recknor C., Mesenbrink P., Bucci-Rechtweg C., Benhamou C.L. (2009). Zoledronic acid for the prevention of bone loss in postmenopausal women with low bone mass: a randomized controlled trial. Obstet. Gynecol..

[bb0130] McClung M.R., Grauer A., Boonen S., Bolognese M.A., Brown J.P., Diez-Perez A. (2014). Romosozumab in postmenopausal women with low bone mineral density. N. Engl. J. Med..

[bb0135] McNabb B.L., Vittinghoff E., Schwartz A.V., Eastell R., Bauer D.C., Ensrud K. (2013). BMD changes and predictors of increased bone loss in postmenopausal women after a 5-year course of alendronate. J. Bone Miner. Res..

[bb0140] Naylor K.E., Jacques R.M., Peel N.F., Gossiel F., Eastell R. (2016). Response of bone turnover markers to raloxifene treatment in postmenopausal women with osteopenia. Osteoporos. Int..

[bb0145] Qaseem A., Forciea M.A., McLean R.M., Denberg T.D., Clinical Guidelines Committee of the American College of Physician (2017). Treatment of low bone density or osteoporosis to prevent fractures in men and women: a clinical practice guideline update from the American College of Physicians. Ann. Intern. Med..

[bb0150] Quandt S.A., Thompson D.E., Schneider D.L., Nevitt M.C., Black D.M., Fracture Intervention Trial Research Group (2005). Effect of alendronate on vertebral fracture risk in women with bone mineral density T scores of-1.6 to −2.5 at the femoral neck: the Fracture Intervention Trial. Mayo Clin. Proc..

[bb0155] Ravn P., Clemmesen B., Riis B.J., Christiansen C. (1996). The effect on bone mass and bone markers of different doses of ibandronate: a new bisphosphonate for prevention and treatment of postmenopausal osteoporosis: a 1-year, randomized, double-blind, placebo-controlled dose-finding study. Bone..

[bb0160] Reid I.R., Brown J.P., Burckhardt P., Horowitz Z., Richardson P., Trechsel U. (2002). Intravenous zoledronic acid in postmenopausal women with low bone mineral density. N. Engl. J. Med..

[bb0165] Reid I.R., Horne A.M., Mihov B., Stewart A., Garratt E., Wong S. (2018). Fracture prevention with zoledronate in older women with osteopenia. N. Engl. J. Med..

[bb0170] Sanchez-Riera L., Wilson N. (2017). Fragility fractures & their impact on older people. Best Pract. Res. Clin. Rheumatol..

[bb0175] Siris E.S., Simon J.A., Barton J.P., McClung M.R., Grauer A. (2008). Effects of risedronate on fracture risk in postmenopausal women with osteopenia. Osteoporos. Int..

[bb0180] Siris E.S., Baim S., Nattiv A. (2010). Primary care use of FRAX: absolute fracture risk assessment in postmenopausal women and older men. Postgrad. Med..

[bb0185] Sterne J.A., Sutton A.J., Ioannidis J.P., Terrin N., Jones D.R., Lau J. (2011). Recommendations for examining and interpreting funnel plot asymmetry in meta-analyses of randomised controlled trials. BMJ..

[bb0190] USPSTF, Curry S.J., Krist A.H., Owens D.K., Barry M.J., Caughey A.B. (2018). Screening for osteoporosis to prevent fractures: US Preventive Services Task Force Recommendation Statement. JAMA.

[bb0195] Wang G., Sui L., Gai P., Li G., Qi X., Jiang X. (2017). The efficacy and safety of vertebral fracture prevention therapies in post-menopausal osteoporosis treatment: which therapies work best? A network meta-analysis. Bone Joint Res..

[bb0200] World Health Organization (2007). WHO Scientific Group on the Assessment of Osteoporosis at Primary Health Care Level.

[bb0205] Wu J., Zhang Q., Yan G., Jin X. (2018). Denosumab compared to bisphosphonates to treat postmenopausal osteoporosis: a meta-analysis. J. Orthop. Surg. Res..

[bb0210] Yang X.C., Deng Z.H., Wen T., Luo W., Xiao W.F., Zhao R.B. (2016). Network meta-analysis of pharmacological agents for osteoporosis treatment and fracture prevention. Cell. Physiol. Biochem..

[bb0215] Yang Y., Luo X., Xie X., Yan F., Chen G., Zhao W. (2016). Influences of teriparatide administration on marrow fat content in postmenopausal osteopenic women using MR spectroscopy. Climacteric..

[bb0220] Yen M.L., Yen B.L., Jang M.H., Hsu S.H., Cheng W.C., Tsai K.S. (2000). Effects of alendronate on osteopenic postmenopausal Chinese women. Bone..

[bb0225] Zhang L., Pang Y., Shi Y., Xu M., Xu X., Zhang J. (2015). Indirect comparison of teriparatide, denosumab, and oral bisphosphonates for the prevention of vertebral and nonvertebral fractures in postmenopausal women with osteoporosis. Menopause.

[bb0230] Zhou J., Ma X., Wang T., Zhai S. (2016). Comparative efficacy of bisphosphonates in short-term fracture prevention for primary osteoporosis: a systematic review with network meta-analyses. Osteoporos. Int..

