# Supplementary material for: Pharmacologic intervention for prevention of fractures in osteopenic and osteoporotic postmenopausal women: Systemic review and meta-analysis
Source: Bone Rep. 2020 Oct 27;13:100729. doi: 10.1016/j.bonr.2020.100729 (PMC7645632; doi:10.1016/j.bonr.2020.100729)
Supplement: Supplemental Fig. 1 — PRISMA flow diagram for study selection. [file mmc1.docx]

**Supplemental Figure 1. PRISMA flow diagram of study selection**

Full-text articles excluded, (n = 124)

Reasons:

- Study objective not consistent with our aim (n = 31)
- No outcome of interest (n = 8)
- Contained women without low bone mass (n = 35)
- Contained women with baseline vertebral fracture (n = 36)
- Duplicate or extension of the same trial (n = 14)

Records excluded after screening the titles and abstracts (n = 1135)

Records after duplicates removed
(n = 1279)

Studies included in quantitative assessment (meta-analysis)
(n = 14)

Studies included in qualitative assessment
(n = 20)

Full-text articles assessed for eligibility
(n = 144)

Records screened
(n = 1279)

Additional records identified using other sources
(n = 12)

## Identification

## Eligibility

## Included

## Screening

Records identified through a database search
(n = 2942)
