# Supplementary material for: Pharmacologic intervention for prevention of fractures in osteopenic and osteoporotic postmenopausal women: Systemic review and meta-analysis
Source: Bone Rep. 2020 Oct 27;13:100729. doi: 10.1016/j.bonr.2020.100729 (PMC7645632; doi:10.1016/j.bonr.2020.100729)
Supplement: Supplementary file 2 — Supplementary material 1 [file mmc2.docx]

**Supplemental Table 1.** Sensitivity Analysis of Bisphosphonates in Meta-Analysis

|  | **First Author (Year)** | **Statistics With Study Removed** | | | | |
| --- | --- | --- | --- | --- | --- | --- |
|  |  | **Points** | **Lower Limit** | **Upper Limit** | ***Z* Value** | ***P* Value** |
| Vertebral fracture | |  |  |  |  |  |
|  | Siris (2008) | 0.51 | 0.35 | 0.73 | -3.61 | <.001 |
|  | Quandt (2005) | 0.53 | 0.37 | 0.75 | -3.54 | <.001 |
|  | Cummings (1998) | 0.34 | 0.15 | 0.78 | -2.55 | .011 |
| Lumbar spine BMD - 1 year | | |  |  |  |  |
|  | Grey (2014) | 4.49 | 3.73 | 5.25 | 11.55 | <.001 |
|  | Grey (2009) | 4.39 | 3.64 | 5.14 | 11.48 | <.001 |
|  | McClung (2009) | 4.61 | 3.75 | 5.47 | 10.49 | <.001 |
|  | Reid (2002) | 4.42 | 3.66 | 5.19 | 11.31 | <.001 |
|  | McClung (2014) | 4.44 | 3.68 | 5.19 | 11.49 | <.001 |
|  | McClung (2006) | 4.35 | 3.60 | 5.10 | 11.35 | <.001 |
|  | Ascott-Evans (2003) | 4.32 | 3.57 | 5.07 | 11.31 | <.001 |
|  | Downs (2000) | 4.37 | 3.61 | 5.12 | 11.34 | <.001 |
|  | Yen (2000) | 3.96 | 3.48 | 4.45 | 16.04 | <.001 |
|  | Cummings (1998) | 4.53 | 3.40 | 5.66 | 7.86 | <.001 |
|  | Bock (2011) | 4.44 | 3.68 | 5.19 | 11.53 | <.001 |
|  | Ravn (1996) | 4.60 | 3.75 | 5.45 | 10.63 | <.001 |
| Total hip BMD - 1 year | | |  |  |  |  |
|  | Grey (2014) | 2.83 | 1.96 | 3.69 | 6.43 | <.001 |
|  | Grey (2009) | 2.86 | 2.02 | 3.71 | 6.63 | <.001 |
|  | McClung (2009) | 2.99 | 1.85 | 4.14 | 5.12 | <.001 |
|  | McClung (2014) | 2.57 | 1.97 | 3.16 | 8.44 | <.001 |
|  | McClung (2006) | 3.01 | 2.11 | 3.91 | 6.56 | <.001 |
|  | Cummings (1998) | 3.14 | 2.21 | 4.06 | 6.65 | <.001 |
|  | Bock (2011) | 3.16 | 2.28 | 4.05 | 6.98 | <.001 |
| Lumbar spine BMD - 2 year | | |  |  |  |  |
|  | Grey (2014) | 5.65 | 5.03 | 6.28 | 17.73 | <.001 |
|  | Grey (2009) | 5.23 | 4.72 | 5.74 | 20.07 | <.001 |
|  | McClung (2009) | 5.00 | 4.99 | 5.01 | 1632.32 | <.001 |
|  | Cummings (1998) | 5.65 | 5.03 | 6.28 | 17.73 | <.001 |
| Total hip BMD - 2 year | | |  |  |  |  |
|  | Grey (2014) | 3.38 | 3.12 | 3.65 | 25.11 | <.001 |
|  | Grey (2009) | 3.75 | 3.09 | 4.41 | 11.20 | <.001 |
|  | McClung (2009) | 3.88 | 2.85 | 4.91 | 7.37 | <.001 |
|  | Cummings (1998) | 4.04 | 3.40 | 4.68 | 12.44 | <.001 |

Abbreviation: BMD, bone mineral density

**Supplemental Figure 2.** Funnel Plots for Bone Mineral Density at the Lumbar Spine at Year 1

**Supplemental Figure 3.** Quality Assessment

A Risk of bias graph.

B Risk of bias summary.
